# Assessment of strategies for male involvement in the prevention of mother-to-child transmission of HIV services in Blantyre, Malawi

**DOI:** 10.3402/gha.v6i0.22780

**Published:** 2013-12-16

**Authors:** Alinane Linda Nyondo, Adamson Sinjani Muula, Angela Faith Chimwaza

**Affiliations:** 1 Department of Community Health, College of Medicine, University of Malawi, Blantyre, Malawi; 2 Kamuzu College of Nursing, University of Malawi, Blantyre, Malawi

**Keywords:** male involvement, HIV and AIDS, PMTCT

## Abstract

**Background:**

Despite the documented benefits of prevention of mother-to-child transmission (PMTCT) of human immunodeficiency virus (HIV) services, the uptake remains low in sub-Saharan Africa. The lack of male involvement (MI) may be one of the reasons for this. However, there are limited data on strategies for MI in PMTCT.

**Objective:**

The objective of this study was to identify strategies that may promote MI in PMTCT services in antenatal care (ANC) services in Blantyre, Malawi.

**Study design:**

An exploratory qualitative study was conducted from December 2012 to January 2013 at South Lunzu Health Centre (SLHC) in Blantyre, Malawi. It consisted of six face-to-face key informant interviews (KIIs) with healthcare workers and four focus group discussions (FGDs) with 18 men and 17 pregnant women attending ANC at SLHC. The FGDs were divided according to sex and age. All FGDs and KIIs were digitally recorded and simultaneously transcribed and translated verbatim into English. Data were analyzed using thematic content analysis.

**Results:**

Three major themes with several subcategories emerged. Theme 1 was a gatekeeping strategy with two subcategories: (1) healthcare workers refusing service provision to women accessing antenatal clinic without their partners and (2) women refusing ANC attention in the absence of a partner. Theme 2 comprised extending invitations and had six subcategories: (1) word of mouth, (2) card invites, (3) woman's health passport book invites, (4) telephonic invites, (5) use of influential people, and (6) home visits. Theme 3 was information education and communication, such as health education forums and advertisements. Of all the strategies, an invitation card addressed to the male partner was most preferred by study participants.

**Conclusions:**

There are several strategies by which men may be involved in PMTCT. Healthcare workers should offer a pregnant woman all strategies available for MI for her to select the appropriate one. Further research and consultations with men should continue to achieve higher levels of MI.

Prevention of mother-to-child transmission (PMTCT) of HIV is the main way of reducing the rate of pediatric HIV infection (1–3). Despite the documented importance and increased benefits of PMTCT services, the World Health Organization (WHO) in 2007 estimated that uptake in sub-Saharan Africa was as low as 11%; it ranged from 8 to 54%, showing the difference in levels of uptake of the service in the region (4). One of the reasons for the low uptake of PMTCT services (e.g. the low numbers of pregnant women undertaking an HIV test and also the low uptake of interventions such as antiretroviral therapy [ART] for PMTCT) is the lack of or the low levels of male involvement (MI) in the services (5–7). Lack of MI contributes to women dropping out of a PMTCT program (6–8).

Male participation in PMTCT improves the uptake and promotes positive attitudes toward the service (6–8). MI in PMTCT is considered crucial in a family setting where men are the main decision makers, as is the case in most African countries (5). Studies have stated that a household head, who in most cases is the husband, greatly influences the woman's ability to seek healthcare or implement health practices and interventions, and studies further report that male partners have a role in the woman's risk of acquiring HIV and also in her uptake of HIV testing and mother-to-child transmission (MTCT) prevention programs (9–11). A Kenyan study reported that involvement of male partners in HIV testing is necessary in increasing uptake of interventions for reducing perinatal HIV transmission. The success of PMTCT of HIV thus depends on cooperation between partners because a woman's partner has a strong influence on the woman's uptake of voluntary counseling and testing (VCT) (9).

PMTCT of HIV services in Malawi are offered within the Maternal and Child Health (MCH) Service and are mostly utilized by women. The uptake of the services in Malawi has progressed steadily. It was estimated that between 52 and 66% of the pregnant women in Malawi had an HIV test in 2009 and 2010, respectively (12), and that between 15 and 23% received effective antiretrovirals (ARVs) for PMTCT. The estimates further indicated that in 2010, Malawi achieved 6% toward the goal of providing ARVs for PMTCT to 90% of HIV-infected pregnant women (13).

Several strategies that can be used for MI in PMTCT have been documented in the literature, such as inviting men through letters or through antenatal cards (14–18), provision of a male-friendly environment that ensures privacy (19, 20), community-based programs (20), male education (14, 18, 21), couple counseling (22, 23), and mass media campaigns (20, 24–26). The rates of MI in PMTCT remain low in Malawi, with reports of 23% of antenatal women being accompanied by their partners in a non-randomized study in Balaka (27), while in Blantyre the rate was 5.2% (28). A study conducted in Malawi noted that strategies available in Malawi were not sustainable because they depended on several assumptions. The strategies, as reported by Kululanga et al. (29), were (1) healthcare provider initiative, (2) partner notification by word of mouth, (3) couple initiative, and (4) community mobilization. It was recommended that lasting strategies be identified for MI that are not dependent on incentives, invitation by a woman, or coercion (29). Despite the reported positive impact of MI on healthcare use and adherence to health interventions by women, there have been no consistent strategies implemented to include them (30, 31).

Currently, there are limited data to show culturally acceptable strategies for MI in PMTCT by the communities involved in PMTCT. To the best of our knowledge, there has not been a study that has identified strategies for MI in PMTCT from multiple sources such as healthcare workers, pregnant women, and men in Blantyre, Malawi. An understanding of the way that men may be involved in PMTCT services may improve the rates of uptake and adherence to PMTCT regimens. The objective of this study was to identify strategies that may be used for MI of PMTCT in Blantyre, Malawi. The study was guided by the **P**redisposing, **R**einforcing and **E**nabling **C**onstructs in **E**ducational/Environmental **D**iagnosis and **E**valuation, **P**olicy, **R**egulations and **O**rganization **C**onstructs in **E**ducational and **E**nvironmental **D**evelopment (PRECEDE–PROCEED) model. It is a planning model that reinforces a participatory approach (32–34). This model was appropriate for the study because it allowed for the identification of strategies for MI together with the people affected who are also service users.

## Methods

As part of a formative study, an exploratory qualitative study was conducted in Blantyre, Malawi, from December 2012 to January 2013 prior to an interventional study. A total of six key informant interviews (KIIs) and four focus group discussions (FGDs) were conducted. Selection of the method was based on its ability to offer an opportunity to find out people's detailed feelings and experiences from their own perspective. It also offered understanding of how people define different behaviors or events and how those are contextually influenced in their setting (35).

### Setting

The formative study was conducted in Blantyre District in the southern part of Malawi. Blantyre has a population of 1,001,984, consisting of 501,000 males and 500,984 females as of 2008; and it comprises urban, semi-urban, and rural areas (36). The district has 32 health centers. The reported HIV prevalence in 2011 among pregnant women in Blantyre was 10.4% (37). The specific health center that was used in the formative study was South Lunzu Health Centre (SLHC), which is 11 km from Blantyre city center and located in the south-eastern part of Blantyre District. The SLHC was selected because of its semi-urban status because that offers a less mobile population for intervention purposes. Furthermore, the semi-urban status offers opportunities to gather views that may be applicable to both rural and urban settings. It has a catchment population of 77,403 as of the 2008–2009 census (36). SLHC provides the following services: outpatient, HIV counseling and testing, family planning, directly observed treatment for tuberculosis, and maternity services, including antenatal care, labor and delivery, and postpartum services. The SLHC has the following categories of health personnel: medical assistants, nurse-midwives, community health nurse-midwife technicians, and health surveillance assistants.

### Data collection

Participants in the FGDs were conveniently sampled with maximum variation in mind. The KIIs were purposely selected according to their knowledge and responsibilities regarding PMTCT of HIV services. A total of six KIIs were conducted with healthcare workers; two nurse-midwife technicians, one medical assistant, two HIV testing and counseling counselors, and one PMTCT coordinator. All key informants attended only one individual interview, which lasted for 45–75 minutes. The interviews followed an in-depth interview guide (see Supplementary file). Additionally, during the same period, four FGDs were conducted with men identified from the clinic and the catchment area and pregnant women who were identified from the antenatal clinic. The groups for FGDs were divided according to age and sex, which translated into two male and two female FGDs. The FGDs followed an FGD guide (see Supplementary file). The FGDs lasted for about 60–90 minutes, depending on the group interaction. In total, there were 17 pregnant women and 18 men involved in the FGDs, and they were further divided as follows: (1) a younger female group, which had an age bracket of 18–24 and 9 participants; (2) an older female group, which had an age bracket of 25 and older, and had 8 participants; (3) a younger male group, which had an age bracket of 18–24 and had 8 participants; and (4) an older male group, which had an age bracket of 25 and older, and had 10 participants. The interviews were conducted by the first author Nyondo with assistance from two research assistants in a private room within the SLHC. Prior to data collection, the research assistants, one male and one female, were trained on the protocol and data collection methods. Participants in FGDs were identified by numbers. All FGDs and KIIs were digitally recorded, and then transcribed and translated verbatim into English concurrently by the researcher. The research assistants checked for completeness of the transcripts and made the final proof reading of the transcripts against the recorded interviews.

### Ethical considerations

Ethical clearance was obtained from the University of Malawi's College of Medicine Research and Ethics Committee (COMREC), and institutional permission was granted by Blantyre District Health Office (DHO). Participation was voluntary. Written informed consent was obtained from all study participants prior to the interviews. Interviews were conducted in a private room. Participants were identified by codes and not their names. Pregnant women and men who declined participation in the study were assured that their decision will not affect receipt of their regular medical care at the SLHC.

### Data analysis

Data were analyzed and managed using NVivo version 9.0. Thematic content analysis was employed as it allows flexibility with analysis of qualitative data because of its non-dependence on a pre-existing theoretical framework (38). Themes which were identified and emerged from the data were inductively coded, while others were deductively derived from the interview guide, objectives, and conceptual framework of the study. Initial transcripts were coded by the first author and another qualitative researcher as a measure for quality control using NVivo 9.0. Each code was then examined for further subcategories, and later codes were organized under commonly recurring themes. Interpretation of themes was realized by reading and rereading the transcripts as well as by verifying with literature and colleagues. The developed themes were verified repeatedly against the digital recordings.

## Results

### Demographic characteristics of the study participants in the FGDs

Table 1 describes the demographic characteristics of the participants in the FGDs. About half of the participants in the FGDs were males (*n*=18). The largest tribal group was Lomwes (14); 11 belonged to the Roman Catholic Church. Many participants (*n*=19) were educated to secondary school level, as were their partners (*n*=20), and half of the participants (*n*=17) were self-employed, while their partners were either formally employed or self-employed (*n*=13 in each case). The mean age for the female participants was 24.7, while for the male participants it was 26.9. The mean gravidity was 2.8, with a range of 1–8. Six male participants had a child younger than 1 year of age.

**Table 1:** Demographic characteristics of the study participants in the FGDs (*N*=35)

Variable	Male	Female
Sex	18	17
Mean age	26.9	24.7
Religion		
Roman Catholic	4	7
Muslim	1	2
Seventh-day Adventist	3	0
Pentecostal	5	4
Other	5	4
Tribe		
Yao	2	5
Ngoni	3	4
Lomwe	8	6
Other	5	2
Education level		
Primary	5	9
Secondary	12	7
College	1	1
Employment status		
Not employed	0	14
Formal	3	1
Self-employed	15	2
Partner's education level		
Primary	11	2
Secondary	6	14
College	1	1
Partner's employment status		
Formal employment	2	11
Self-employed	7	6
Not employed	9	0
Age of youngest child (males only)		
Pregnant wife	4	
Under 1	6	
1– < 2 years old	1	
2– < 3 years old	2	
3– < 4 years old	3	
4– < 5 years old	2	

Mean gravidity: 2.8, range 1–8.

### Demographic characteristics of the participants in the KIIs

Demographic characteristics of the participants in the KIIs were as follows: Five of the respondents were females, and only one was male. Four of the six were married, one was single, and one was widowed. In terms of religion, two were Presbyterians, two were Seventh-day Adventist members, one was a Baptist, and one was a Roman Catholic. Three participants were Ngonis by tribe, and one was Chewa, one Yao, and one Lomwe. Two of the six participants were educated to secondary school level, and four were educated to college level. Four of the respondents had worked for less than 5 years, while one had worked for 6–10 years and another for 10–15 years. The mean age for KII participants was 31.8.

### Proposed strategies for male involvement in PMTCT of HIV services

Three major themes emerged from the discussion in relation to strategies for MI in PMTCT of HIV services. These were (1) a gatekeeping strategy, (2) extending invitations, and (3) information education and communication. The three themes had several categories as described in Table 2.

**Table 2:** Proposed strategies for male involvement in PMTCT of HIV services

Theme	Categories
Theme 1: gatekeeping strategy	*Refusing a woman medical (antenatal) attention*
	*Refusal of medical (antenatal) care by the woman*
Theme 2: extending invitations	*Woman inviting her partner*
	*Partner notification slip (invitation card)*
	*Writing in the woman's health passport book*
	*Telephonic invites*
	*Using influential people or people in authority*
	*Home visits*
Theme 3: information, education, and communication	Radio messages
	Adverts
	Motivation talks

### Theme one: a gatekeeping strategy

There were two strategies for MI proposed under the gatekeeping theme. The proposed strategies may be implemented by health workers, pregnant women, or traditional authorities as follows.

#### 2.4.1 Category one: refusing a woman medical attention

Some respondents in the younger male group suggested that medical personnel should withhold medical attention to a woman unless she is accompanied by her male partner.

I think a woman should not receive any medical help until her husband comes. (Younger male [YM] FGD-)

However, there were other respondents within the same group who objected to that strategy because they regarded it as unacceptable. One young male participant did not support use of that strategy because he feared that in an emergency situation, health workers are obliged to offer medical attention. Additionally, it was stated that this strategy would not be practical in instances where a man in not available in the area or country:

I do not agree with Number 7 because if a woman is having problems and is in need of medical attention, the health workers will not wait for the husband to come. … For instance there are some women who will tell you that they were impregnated by their husbands but the husband is in Jo'burg [Johannesburg], the healthcare workers will not leave her unattended because of that. (YMFGD)

#### 2.4.2 Category two: refusal of medical care by the woman

Respondents from younger female focus group suggested that a woman should unilaterally decide against antenatal care (ANC) services unless her husband accompanies her. Although there was a belief that some men need drastic measures, such as a woman's non-attendance or non-initiation of ANC, in order for a male partner to be involved in PMTCT services, the respondents also noted that some men may not avail themselves following word-of-mouth invites alone. The following quote illustrated this category:

[T]elling him that one [a wife] will not go unless he accompanies the wife. He will know that, if his wife is refusing initiating antenatal care, then it is true that they really need a partner so he may ponder over accompanying his wife. … Because just by word of mouth by asking him to accompany you [the wife] for antenatal care, he may refuse, such that when he says he does not want, we will give up because we know that even if we talk over it, he will not come along, and we just come without him but by telling him that one will not go unless the couple goes together [unless we go together] maybe it may help. (Younger female [YF] FGD)

The method may be, when you have been asked for an HIV test, as a woman, you inform the man that a woman may not proceed with other activities until he accompanies her for both of them to be tested [of HIV], maybe using that method, most men may accept and come for the visit. He needs to know that his wife may not be helped or assisted at the clinic if he does not accompany her. (YF FGD)

### Theme two: extending invitations

The participants in both KIIs and FGDs expressed the need to extend invitations to men for their involvement in PMTCT of HIV services. Several categories emerged under this theme, illustrating the different forms of invites proposed.

#### 2.5.1 Category one: woman inviting her partner

Some respondents from the male's FGD and KIIs expressed that women may invite their partners for PMTCT services. It was reported that a woman should pass on the message that the health worker has given her to her husband. This was expressed as follows:

[A] pregnant woman should be encouraged that at her subsequent visit she should come with a partner. (KII respondent)

[O]n the day that a woman comes to start antenatal care, on that day, if there is an opportunity this woman has to be told this: “[Amayi,] you have commenced antenatal care, and we would like at the next visit to meet your husband. This is information that we cannot give to you alone, we need your husband to be here.” This woman will find means of communicating with her husband; even if her husband may be a difficult man. … It is not difficult for a woman who has reported for antenatal care to bring her husband along at the next visit. A man who is responsible and understanding will come. (Older Male [OM] FGD-)

Some male participants in the FGD and a KII respondent expressed concern over asking women to invite their partners for PMTCT of HIV services. They indicated that a man may not easily believe and act on what a woman has asked him to do. The following quote illustrated this:

This method of using women to get to the man, may not work … relying on the woman to inform the man, it may not work, she may be beaten up. (OM FGD)

Just by word of mouth a man may think it is just his wife who wants him to accompany her and not the health workers because most men believe that a pregnant woman is demanding. It is better in written format or even in the health passport book. (KII respondent)

#### 2.5.2 Category two: partner notification slip or invitation card

Another method that was proposed was the use of a partner notification slip. Respondents from both male and female focus groups agreed that this would be the best method to use because it will be addressed directly to the man, and a woman will just pass on an envelope without explaining the message in the envelope. Male participants expressed that this strategy may be perceived as respectful since the message will be addressed to him:

I think, for a man to believe that the wife's message is true; there should be a written notification. … Not much will be said on the paper, the letter ought to be stamped with a hospital stamp. When the man sees the stamp he will know that he is wanted. (OM FGD)

[I]f possible a card is better, for now we may just write in a health passport book, but in future they should develop cards just like those for sexually transmitted infections [STIs] partner notification slip. Even better would be a general invitation for every pregnant mother, because if we [healthcare workers] indicate issues of HIV status, [it] would lead people to assume that only those whose wives are HIV infected are being invited to the clinic so that when they see that a man has gone to the clinic they will conclude that his wife is HIV infected. (KII respondent)

#### 2.5.3 Subcategory: design of the invitation card (notification slip)

Participants recommended having a hospital stamp on the card as proof that the invite originated from the health center and also suggested that the invite should make no mention of HIV testing. Emphasis was made on uniformity between the information relayed in the invite and the information passed on to the woman in order to avoid arguments that may arise from conflicting messages. The following were the suggestions that came out:

[T]he stamp will be proof enough that it came from the hospital and it will take away all the doubt that maybe someone wrote the note for her. (YM FGD)

I think the man should be invited in this manner “Mr ___:Your wife has started antenatal care, we are therefore asking you to accompany her so that we counsel you together on how you can care for her at home and how you can care for the pregnancy.” Do not add in anything to do with HIV testing. (YM FGD)

Other participants felt that there should be reference to HIV testing on the invite; however, this was rejected by most participants because they said they could foresee the message becoming a barrier in itself to MI. The following quote illustrates this:

Sometimes men do not want to test [for HIV] such that if you include testing [HIV testing in the invitation] he may not come. It is better for him to get to know about testing [HIV testing] while here [at the clinic] after explanations. (YM FGD)

The participants reported some challenges with the use of notification slips. The concerns centered on women who may not want their partners to accompany them or potential loss of the invite.

If the woman does not want [her partner to accompany her], she will tear it off. (YFFGD)

#### 2.5.4 Category 3: writing in the woman's health passport book

Another proposed strategy was to extend the invite to the man by writing in the woman's health passport book and asking the woman to show it to her partner when she gets home. It was stated that this method is easy because every antenatal woman has a health passport book that she brings to every antenatal visit.

In the health passport book, they may believe it because no one can write in the book anyhow. … While on a piece of paper he may think that you asked someone to write it for you, [he may think] maybe you have a boyfriend at the clinic. (Older female [OF] FGD)

Male participants from FGDs opposed the strategy of inviting them for PMTCT of HIV services using the woman's health passport book. They were of the opinion that the health passport book belongs to the woman. This was discussed in comparison to the notification slip:

It needs to be an invite to the man and not in the woman's health passport book! As long as it is in the health passport book then it will be a woman's thing. (OM FGD)

#### 2.5.5 Category 4: telephonic invites

It was also suggested that a healthcare worker should request the male partners’ phone number from his pregnant wife and make a call extending an invitation to the male partner while the woman is still at the clinic. The following quotes suggest this:

That [telephone call] is a good method especially if the call is made while the wife is still at the clinic and informs him that his partner is at the clinic, he will be alarmed and will rush to come over here. (YF FGD)

There is technology these days; we may get his phone number … and ask for air time from the district health office (DHO), so we may use that airtime to call this man … and tell him “your wife was here she came for antenatal care and I noted that we cannot proceed [with antenatal care] and I would like you to come so that we discuss with both of you as a couple.” (KII respondent)

Respondents from the older male and older female FGDs had reservations on using telephonic invites as a strategy for MI in PMTCT of HIV services. Participants expressed fear that some male partners may not believe that the invite came from the clinic, which may trigger quarrels between the partners. The other observation was that not all men have phones or access to a phone, thereby limiting the number of men reached with this strategy. The following quotes illustrate this.

A phone call is not a good method because the call is made only once and there is nothing that will serve as a reminder to this man … not all men have cell phones; a letter would be the best option. (OM FGD)

He will think that one arranged with a friend, maybe you [a pregnant wife] once asked him to accompany you and he refused the first time. Maybe you now want that he should accompany you, you may easily ask a friend to call him and tell him that he is needed at the clinic at a specific time. He may say “just because I refused the other time, you asked a friend so that I escort you, I will not even go there”. (OF FGD)

#### 2.5.6 Category 5: using influential people or people in authority

A strategy of using chiefs or other influential people was advocated for by older male participants in the FGDs. They stressed that chiefs or influential people have the capacity to ask men to be involved in PMTCT of HIV services and that men will likely respond positively to the request.

If the government ensured that women are delivering at the hospital and not at the traditional birth attendants; they may do the same in this case. … I think this may not be a problem to be effected because a chief has his own people whom he oversees. … If the chief gives a directive that every woman who is pregnant and wants to start antenatal care must report to the clinic with her husband, anyone who does not do that will be fined. … I think it may be easier. If they managed that with traditional birth attendants [TBAs] who were getting money and people were not travelling long distances to get there, and now people come to deliver here [SLHC], why would it be difficult for him [a male partner] to escort his wife? This [chiefs giving out a directive] is an easier method. (OM FGD)

#### 2.5.7 Category 6: home visits

A health worker ought to find the man at his home, and if he is not home then a message stating the return date could be left for the man. The following quotes illustrated this.

Sometimes, it is good to conduct a home visit. One needs to go find a male partner at his home and if the man is not home, you [healthcare provider] leave a message for the return visit. (OM FGD)

However, this proposal was regarded as expensive to implement and also time consuming, as expressed in the quote below:

OK, following them [women and their partners] up because we [(health care providers] have physical address for women and one knows where a specific woman comes from. I would say it may be difficult but it may also not be difficult. It may not be difficult because if the health surveillance assistants [HSAs] have been informed about this programme, then each one usually knows the people in his/her catchment area, and one would know that “this woman is pregnant” and we also need to check the maternity register for her residential address so that each HSA knows the number of pregnant women residing in his/her area. So when one has checked through the register and has identified the women one may be able to reach them at their house respectfully and explain to them the purpose of the visit for them to understand. (KII respondent)

### Theme 3: information, education, and communication

Respondents from KIIs and male FGDs suggested the use of media and health education strategies in order to reach men. The suggested strategies include radio messages, adverts, and motivation talks on MI in PMTCT of HIV services.

We need to educate them [men] on the importance of male involvement. Using different communication methods maybe helpful. … Another method could be radio messages. (KII respondent)

Another good method would be to have some advertisement and add in some attractive messages so that a man is attracted to them. (OM FGD)

In order to find the men without any difficulties like here at this hospital [SLHC], which is patronized by men and women, we need to inform them even before they begin the consultation process in the outpatient department and then explain to them the advantages of men accompanying women for antenatal care [ANC]. This means that the message will be spreading slowly and the service will be taken up. (OM FGD)

## Discussion

The current study aimed at identifying strategies that may be used for MI in PMTCT of HIV services. The differences between the suggested strategies and what is reported in the literature involve the format and specific message to be included in the strategy. Although the strategies differed among women and men, the key findings illustrate the need to use strategies that are male friendly in order to encourage MI in PMTCT.

Female participants suggested extending invites to their partners through the woman's health passport book. This finding was similar to results obtained from studies in Uganda and Tanzania, where invitations written in the spouse's ANC card were advocated for (14, 16, 18). Conversely, men had dissenting views to the use of ANC passbooks because of the potential of demeaning their masculinity because that strategy probably positions them under the woman by virtue of the invite coming through a woman's health passport book. This finding builds upon what was reported by Bwambale in Uganda, where VCT uptake by men showed that men were willing to be tested for HIV as a couple; however, they did not follow through with that because it would be culturally inappropriate for the men to attend VCT services with their partners as it would undermine their masculinity (39).

Male participants preferred an invite in an envelope, which they deemed individualized and respectful, thereby asserting the strong cultural values of a man as the head of a household. A study in Tanzania also showed that men availed themselves following receipt of formal invitations for antenatal services (17). This finding is consistent with what was reported in an Elizabeth Glaser Pediatric AIDS Foundation (EGPAF) program in Zambia and Uganda, where partner notification slips were effective in that the majority of men reported to the clinic, thus presenting an opportunity to involve men in the services (40). Partner notification slips are currently used as a means of inviting men for sexually transmitted infection (STI) treatment in antenatal clinics in Mwanza, Malawi; however, their effectiveness has never been explored regarding men's attendance to PMTCT of HIV services (29). The exact message to be included in a partner notification slip, as reported by this study, is to be more general without mentioning HIV testing (to avoid the latter being a barrier to MI). Studies in other African countries reported men shunning PMTCT services because the services addressed issues on HIV and AIDS, and thereby men disassociated themselves from the service to avoid knowing and disclosing their HIV status (14, 20).

A study done in Mwanza, Malawi, by Kululanga identified another form of partner notification, word of mouth, as a strategy for MI in maternal health services, however, men expressed that this strategy reduced them to beneficiaries and not partners in the service (29). In the current study, word-of-mouth invite was suggested; however, the participants also noted that it may predispose a woman to being abused. This finding is consistent with the finding by Burke et al. in Tanzania that suggested that the traditional way of using women to relay health information to males was a barrier to MI because men preferred to receive the message through other men (41).

Use of influential people in a community such as chiefs and other leaders is a strategy reported in the current study for MI in PMTCT. Community members respect their leaders and are likely to adopt practices promoted by leaders out of reverence, subordination, or fear. This finding builds on the findings by Kululanga et al. in Mwanza and by Kasenga in Thyolo, Malawi, where community leaders served as advocates for MI (29, 42). However, the male community in Mwanza described this strategy as punitive because women who reported for ANC alone were refused ANC (43). Reports from the second reproductive health conference in Lilongwe, Malawi, advocated for the involvement and support of traditional leaders in mobilizing their communities and in encouraging male participation in the maternal health issues of their partners (44).

In this study, the participants suggested denying a woman access to ANC as a strategy for MI, even though they were also cognizant that it would infringe on the woman's right to medical care. Some women proposed that a woman should voluntarily refrain from ANC until her partner accompanies her as a means of MI. There was no literature to substantiate these strategies.

Malawi has health surveillance assistants (HSAs), each overseeing a clearly demarcated catchment area offering various health services (45). An additional component to HSAs’ responsibilities within their communities, as suggested by this study, could be conducting home visits to partners of pregnant women as a way of promoting MI in PMTCT. This finding entails adding MI in PMTCT promotion to the existing home-based door-to-door HIV-testing model that is currently part of the HIV-testing models in Malawi (46). A randomized study done in Kenya showed that home-based HIV testing was accepted and resulted in increased male partner HIV testing than that of partners of pregnant women who were invited by letter for an HIV test to the clinic (47).

Another strategy that was proposed was the use of phones. However, for telephonic messages, participants indicated that this method may not be feasible for some clients because it limits the clientele to be reached by that method and opens up room for mistrust. Furthermore, literature has not reported much on the strategy. One would argue that the other strategies proposed, such as telephonic invites and use of leaders, were not sustainable and depended more on availability of resources, such as having airtime for phone use and assuming that couples have telephone lines for use. Nevertheless, findings reported in this article suggest that MI requires a combination of different strategies as there were different potential strategies proposed.

The current study also suggested that information, education, and communication (IEC) strategies such as radio messages, adverts, and motivation talks on MI in PMTCT would promote MI. Currently, there are media messages on MI in PMTCT; however, their effectiveness has not been assessed. Similarly, in a Tanzanian study, healthcare workers suggested the use of television (TV) programs as a strategy for attracting men into PMTCT programs (24). Additionally, a study in Kenya showed that attendance to media was associated with uptake of HIV testing because women who read newspapers and listened to the radio daily, and men and women who watched TV, took an HIV test more than their counterparts who did not read newspapers or watch TV (48). Male education as a strategy as proposed in this study remains consistent with findings from other African studies, which also suggested male education and sensitization as strategies for incorporating males into the services (14, 18, 21).

Promotion of couple counseling, which has been advocated in most African settings as a way of incorporating males in PMTCT (22, 49), was not suggested as a strategy in this study. Possible reasons for non-emergence of it as a strategy could reflect what Falnes commented: that as much as couple counseling is a good and presumably effective strategy, it may not achieve its intended purpose because it takes place in antenatal clinics, which are perceived to be a woman's domain (25). Another reason was reiterated in a Ugandan study which identified that a major obstacle with couple counseling is the responsibility that it places on a woman to extend the invitation to the male partner (26). The study further reported that a woman with that responsibility may be unwilling to discuss a presumably sensitive issue for fear of potential adverse consequences from her partner (26). Nonetheless, couple counseling is an upcoming strategy for MI as it has been reported that couples are at a high risk of HIV infection and transmission because rarely are preventive behaviors such as condom use practiced within a couple setting (50, 51). A review of studies on couple-centered HIV testing in sub-Saharan Africa revealed that such services, in spite of the documented benefits and acceptability of the service, have not been rolled out on a larger scale (52).

### Strengths and limitations of the study

One of the strengths of this study was that data were collected from both healthcare workers and users of PMTCT services, such as men and pregnant women. The limitation with the study is that this was conducted in one setting in Blantyre and may not adequately reflect the views of other settings within Blantyre District.

## Conclusions

Overall, this study showed various strategies for MI in PMTCT services. The acceptability of MI strategies depends on cultural contexts and other individual intrinsic factors, with men insisting on preservation of their masculine identity even in health issues. The success of MI may require multiple interdependent strategies that are culturally, socially, politically, and gender acceptable in the different communities and for different couples. Healthcare workers should present to a pregnant woman the available strategies for a woman to make an informed decision on a strategy that is suitable for her and her partner. The study showed that most participants preferred a notification slip or invitation card to the male partner as a means of incorporating men into the service.
